# Error in Byline

**DOI:** 10.1001/jamanetworkopen.2023.43869

**Published:** 2023-11-01

In the Original Investigation titled “Survival Outcomes by Race and Ethnicity in Veterans With Nonmetastatic Castration-Resistant Prostate Cancer,”^1^ published October 11, 2023, there was an error in the spelling of the fifth author’s surname. This article has been corrected.^1^
